# Novel platinum bipolar electrode for irreversible electroporation in prostate cancer: preclinical study in the beagle prostate

**DOI:** 10.1038/s41598-021-96734-5

**Published:** 2021-08-25

**Authors:** Bumjin Lim, Hong Bae Kim, Seung Jeong, Song Hee Kim, Jeon Min Kang, Yubeen Park, Dong-Sung Won, Ji Won Kim, Dae Sung Ryu, Yunlim Kim, Jung-Hoon Park, Choung-Soo Kim

**Affiliations:** 1 Departments of Urology, Asan Medical Center, University of Ulsan College of Medicine, 88 Olympic-ro 43-gil, Songpa-gu, Seoul, 05505 Republic of Korea; 2 Department of Biosystems and Biomaterials Science and Engineering, Seoul National University, Seoul, 08826 Republic of Korea; 3 Biomedical Engineering Research Center, Asan Institute for Life Sciences, Asan Medical Center, 88 Olympic-ro 43-gil, Songpa-gu, Seoul, 05505 Republic of Korea

**Keywords:** Biomedical engineering, Urinary tract

## Abstract

The exposure of the prostate to high electric field strength during irreversible electroporation (IRE) has been extensively investigated. Multiple monopolar electrodes, however, have risks of organ piercing and bleeding when placing electrodes. A novel bipolar electrode made of pure platinum and stainless steel was developed for prostate cancer ablation. Voltages of 500 and 700 V were applied to the beagle prostate with this electrode to evaluate ablated tissues and their characteristics. IRE procedures were technically successful in all dogs without procedure-related complications. The current that flowed through the anode and cathode while applying 500 and 700 V were 1.75 ± 0.25 A and 2.22 ± 0.35 A, respectively. TUNEL assays showed that the estimated ablated areas when applying 500 and 700 V were 0.78 cm^2^ and 1.21 cm^2^, respectively. The minimum electric field strength threshold required for induction of IRE was 800 V/cm. The platinum electrode was resistant to corrosion. The IRE procedure for beagle prostates using a single bipolar electrode was technically feasible and safe. The novel bipolar electrode has great potential for treating human prostate cancer with fewer IRE-related complications.

## Introduction

Prostate cancer is the most common cancer diagnosed in men, with an estimated 248,530 newly diagnosed men in the United States in 2021^1^. Of the 3.1 million men newly diagnosed with prostate cancer in 2003–2017, 77% had localized prostate cancer^1^. Randomized clinical trials have shown that, compared with watchful waiting, radical prostatectomy improves overall survival or delays the development of metastatic disease in men with localized prostate cancer^2,3^. Although radical prostatectomy is regarded as the standard treatment, it can cause urinary incontinence and sexual dysfunction, and reduce patient quality of life^4–6^.

The goal of cancer treatment is to minimize side effects while treating the disease. Thus, focal treatment options have been developed to prevent injury to the neurovascular bundle and urinary continence mechanisms^7^. Various ablative procedures have therefore been developed for the focal treatment of prostate cancer. These include high-intensity focused ultrasound, cryotherapy, laser ablation therapy, radiofrequency ablation, photodynamic therapy, and irreversible electroporation (IRE)^8^.

IRE is a modality in which high electric field strength is focused to ablate prostate tissues. The electric fields between two monopolar electrodes inserted into tissues cause tissue ablation, including the generation of irreversible nano-sized pores on the surfaces of cell membranes^9^. These irreversible pores cause cells to undergo either catabolic or anabolic apoptosis, followed by necrosis^10^. Unlike other focal therapies, this property of IRE can preserve blood vessels and neurons surrounding cells, killing only the involved cells. Thus, one important advantage of IRE is the rapid recovery from injury^9,11,12^. Because the prostate is located far from the heart, IRE can be used to ablate prostate cancer tissues, while avoiding its heart-associated drawbacks, including muscle contraction and arrhythmia^12^.

The application of IRE to prostate cancer requires two or more electrodes. Multiple electrodes are utilized and are required to be placed in a parallel position to meet the requirements for ablating a region wide enough to remove the entire tumor. Increasing the number of electrodes not only increases costs but can increase IRE-related complications such as piercing and bleeding, as well as requiring a high level of technical skill for accurate penetration. Patients who are suitable for focal treatment of prostate cancer are those with smaller tumors. Reducing the number of electrodes may be more efficient if the cautery range is adequate. Bipolar electrodes each have a single needle containing an anode and cathode^13^, reducing the number of electrodes required for treatment. It is also easy to plate the electrode inserts. Although bipolar electrodes are made of medical grade stainless steel, electrochemical corrosion can occur on their surfaces during electroporation. These electrochemical reactions due to corrosion are toxic to tissues^14^. Bipolar electrodes have recently been used in animal studies^14–16^.

We recently developed a novel platinum bipolar electrode that can be used for single-electrode ablation while avoiding corrosion of the electrode. We hypothesized that the use of a single bipolar electrode could reduce the risk of IRE-related complications with reducing the number of penetration of the prostate, particularly perforation or bleeding during the IRE procedure. The present study investigated the efficacy and technical feasibility of IRE using the newly developed bipolar electrode in a prostate cancer model in beagle dogs.

## Materials and methods

### Irreversible electroporation devices

**Figure 1:**
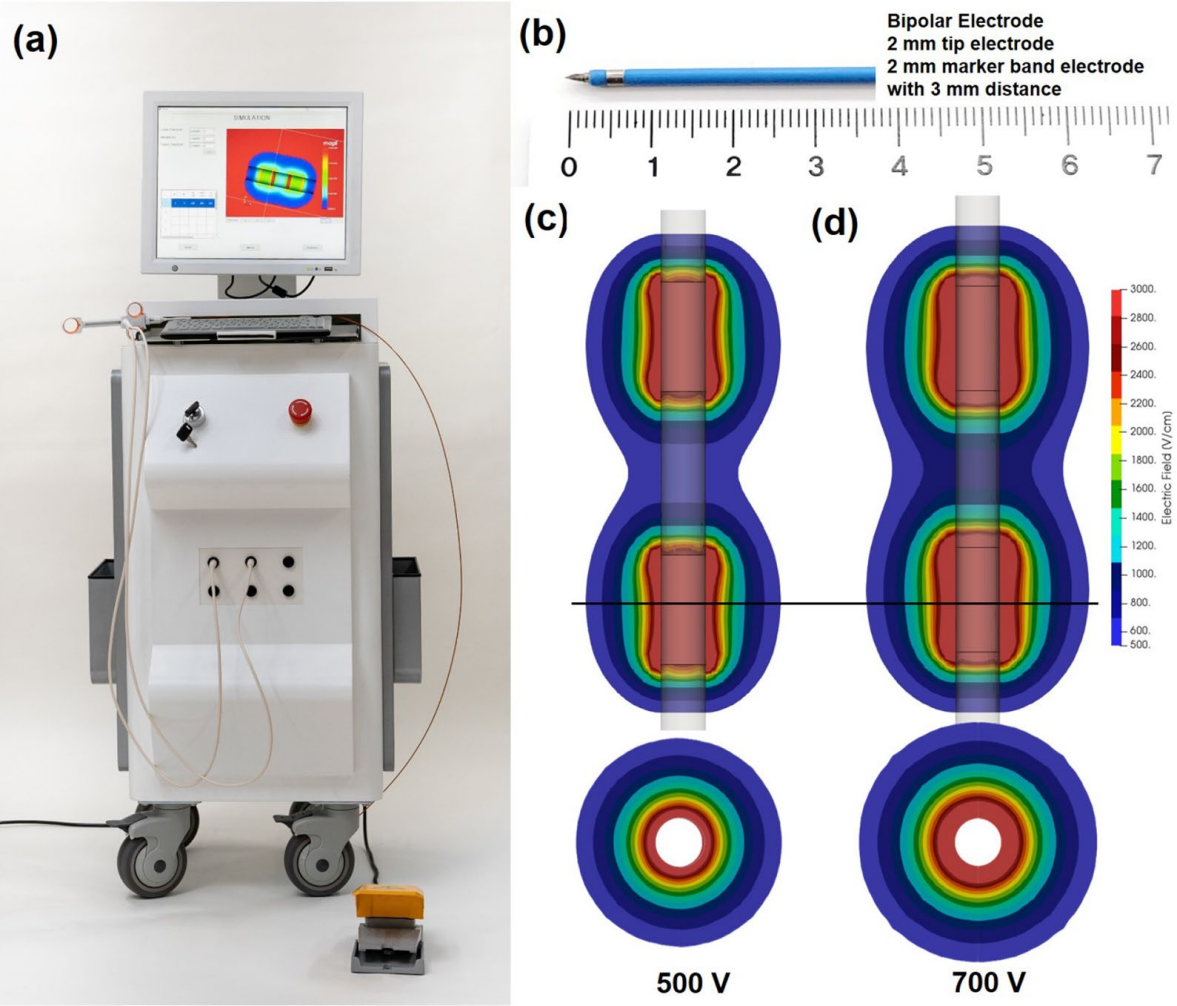
Experimental setup for irreversible electroporation (IRE) of the beagle prostate glands. (**a**) Photograph of the IRE system. (**b**) Photograph of the bipolar electrode, which was composed of a 2 mm electrode tip (stainless steel) and a 2 mm marker band (platinum) located at a distance of 3 mm. (**c**,**d**) Simulation of the distribution of electric field strengths during application of (**c**) 500 V and (**d**) 700 V in the horizontal and vertical directions between the electrodes.

The bipolar electrode Bi-E-EPO1 was obtained from The Standard Co. Ltd. (Gyeonggi-do, Korea). This electrode, which was made of a platinum alloy (palladium:platinum 1:9) at the marker band portion and stainless steel at the tip portion, had a diameter of 1 mm, a distance between the centers of the electrodes of 5 mm, and a band width of 2 mm. The electrode was connected to the IRE equipment (EPO1, The Standard, Co. Ltd.), which is capable of applying an electric field of amplitude up to 3 kV at pulse widths of 100–1000 μs and at pulse intervals of 100–2000 μs (Fig. 1a,b).

### Animal study design

The protocol for the animal study was approved by the Institutional Animal Care and Use Committee of the Asan Medical Center (IACUC-2020-14-277) and conformed to the US National Institutes of Health guidelines for handling of laboratory animals. The study was carried out in compliance with the ARRIVE guidelines. Three certified-healthy male beagle dogs, weighing 14–16 kg at 12 months of age, were inspected and quarantined for 7 days after procurement, according to the health monitoring report provided by the supplier. These dogs were confirmed to be healthy and suitable for experiments after monitoring during the 7 day acclimatization period. Subsequently, the animals were maintained in individual cages over 0.74 m^2^ in area, at a temperature of 23 ± 2 °C, a relative humidity 50 ± 10%, a ventilation frequency of 10–15 times/h, a lighting time of 12 h (on at 8 am and off at 8 pm), and an illumination of 150–300 Lux. Dogs were fed autoclaved food and water.

Of the three male beagle dogs, one was used as a sham control, and the other two were subjected to the IRE procedure. IRE procedures were performed by exposing the left side of the prostate to two voltages, 500 and 700 V, with the right, unexposed side of the prostate serving as a normal control. A digital oscilloscope (TDA3044B, Tektronix, US) was used to measure the electrical current. A hole-type current probe (TCP305A, Tektronix) was clamped to a cord connecting the electrode and the pulse generator.

### Simulation of electric fields

The electric field strength between the electrodes was determined using the Laplace equation. The conductivity of the tissue was hypothesized to be constant. An electric field distribution in the steady-state was determined by solving the equation:

1 $$\nabla^{2} \emptyset = 0$$

where ∅ is the applied electric potential, assuming that the electrode length is greater than the distance between the electrodes. The surface effect of the tissue was neglected. The electric field strength depends on the gradient of the electric potential:

2 $$E = \nabla \emptyset$$

The boundary condition was defined as either ∅ = 0 or *V*_0_ in the applied tissue. The resting boundaries were hypothesized to be electrically insulating ((d∅)/dn = 0). Under these assumption, the equations were solved and coded. This code was connected to the open source software OpenFOAM and developed as a simulation program (The Standard Co. Ltd., Korea). Prior to the IRE procedures, the distribution of electric field strengths was determined using the software (Fig. 1c,d).

### Irreversible electroporation procedure

**Figure 2:**
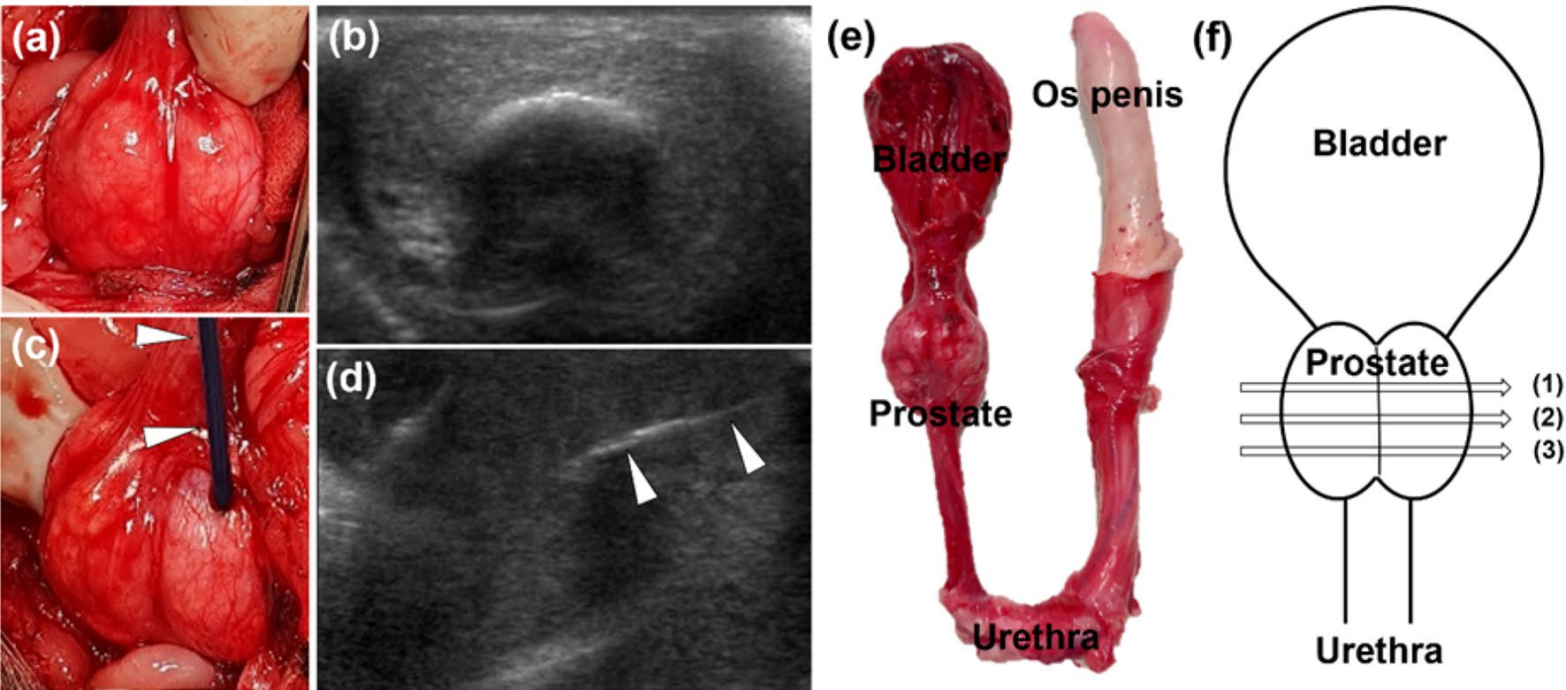
Technical steps in the irreversible electroporation (IRE) procedure using the bipolar electrode and pathologic findings and location of tissue samples examined histologically. (**a**) Photograph and (**b**) ultrasonographic view showing the prostate of a beagle dog before the IRE procedure. (**c**) Photograph and (**d**) ultrasonographic view showing the successful insertion of the bipolar electrode (arrowheads) into the left side of the prostate under ultrasonographic guidance. (**e**) Anatomy of the lower urinary tract of a beagle dog. (**f**) Schematic illustration showing the locations of proximal (1), middle (2), and distal (3) tissue samples ablated by IRE.

General anesthesia was induced in each dog by intramuscular administration of ketamine hydrochloride (Yuhan, Seoul, Korea) and atropine sulfate (Daewon, Seoul, Korea), followed by insertion of an endotracheal tube, and administration of inhaled of 0.5–2% isoflurane (Ifran®; Hana Pharm. Co., Seoul, Korea) mixed 1:1 with oxygen at a rate of 510 mL/kg/min. A longitudinal abdominal incision was made at the midline, and the urinary bladder and urethra were identified. The prostate was subsequently identified by tracing along the urinary tract. In the sham control dog, the bipolar electrode needle was inserted into the left side of the prostate tissue under ultrasonographic guidance and removed after 30 s. In the second dog, the IRE was applied to the left side of the prostate at an electric field strength of 500 V with a pulse width of 100 μs and a pulse interval of 2000 μs. Ten groups of 20 pulses each, for a total of 200 pulses, were applied to avoid the thermal effects of intensive electrical current (Fig. 2a–d). In the third dog, IRE was applied to the left side of the prostate at 700 V and the same pulse width and interval as above. Five groups of 20 pulses each were applied, followed by two separate individual pulses, for a total of 102 pulses.

Therefore, the total electrical energy per volume was 166 J/cm^3^ using the following equation:

3 $$Energy\;per\;Volume\; \left( {{\text{J/cm}}^{3} } \right) = E^{2} \sigma t$$

where *E* is the electric field amplitude, *σ* is the electrical conductivity of the prostate tissue, and *t* is the total pulse duration. The electrical conductivity 0.83 S/m was matched to electric current of 1.75 A when 500 V applied to the canine prostate tissue. Besides, 0.75 S/m was matched to 2.22 A for application of 700 V. The dogs were sacrificed 18 h after the procedure by administering an overdose of xylazine hydrochloride (Rompun; Bayer, Seoul, Korea). The used electrodes were harvested to confirm anodic corrosion and the electrode surface was observed with a reflecting microscope (AM4113TL, Dino-Lite, Hsinche, Taiwan).

### Histologic examination

The dogs were anesthetized 18 h after IRE, and the urethra, prostate, and urinary bladder in each animal were identified by surgical exploration, followed by gross examination to determine possible tissue injuries, including cell apoptosis, during the IRE procedures^10,17^. The prostate in each dog was gently separated from the urethra and bladder. Tissue samples were fixed in neutral buffered formalin for 24 h, and the proximal, middle, and distal portions of the IRE-ablated segment were sectioned transversely (Fig. 2e,f). The specimens were subsequently processed for gross and microscopic examination. The slides were stained with hematoxylin and eosin (H&E) and assessed histologically using a Digital slide scanner (Pannoramic 250 FLASH III, 3D HISTECH Ltd, Budapest, Hungary). Measurements were obtained with a digital microscope viewer (CaseViewer, 3D HISTECH Ltd) and ImageJ software (1.52p, Wayne Rasband, USA).

### Immunohistochemistry

Immunohistochemistry (IHC) was performed on paraffin embedded sections with TUNEL (ApopTag, Qbiogene, Darmstadt, Germany) as the primary antibodies. The sections were visualized with a BenchMark XT IHC automated immunohistochemical stainer (Ventana Medical Systems, Tucson, AZ). Histological evaluation was based on the consensus of three observers who were blinded to the assignment of each animal.

## Results

### Technical outcomes

IRE procedures were technically successful in all dogs without procedure-related complications. All dogs showed mild hematuria immediately following the procedure, but this hematuria spontaneously resolved in all dogs within 1 day. All of the dogs survived until the end of the study without IRE-related complications. Application of IRE to the second and third dogs at 500 and 700 V, respectively, yielded mean electrical currents of 1.75 ± 0.25 A and 2.22 ± 0.35 A, respectively (Supplementary Fig. 1). The corresponding electrode conditions were found to be noble for anodic corrosion of platinum, but slightly darker in stainless steel due to electrochemical corrosion (Supplementary Fig. 2).

### Histological findings

**Figure 3:**
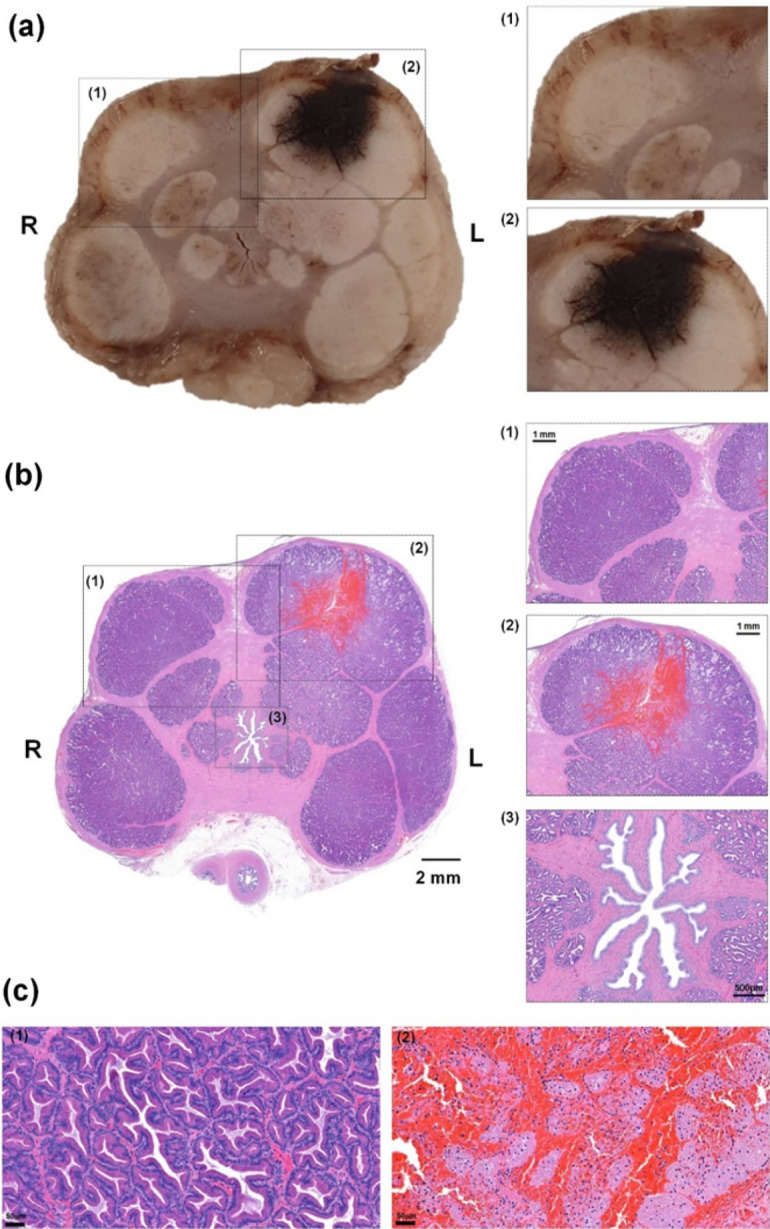
Representative histologic and microscopic images of tissue sections of the beagle exposed to 500 V. (**a**) Gross images with magnification (1, 2) showing necrotic tissue changes and edema on the left side of the ablated prostate. (**b**) Magnified microscopic images showing normal prostate tissue (1), hemorrhage with necrotic changes (2), and the prostatic urethra (3). Histologic staining of an (**c**) intact (1) and an IRE-treated (2) tissue sample.

Overall gross and histologic findings are shown in Supplementary Fig. 3. The ablated regions in the resected tissues were blacker in dogs that underwent IRE than in the sham control. H&E staining showed considerable hemorrhaging in the ablated sections of the prostate. The magnified images showed that the prostatic vesicles in the sham control and the right sides of the prostates in the dogs subjected to IRE were tightly-arranged and full of glandular cavities (Fig. 3a,b). The ablated left sides of the prostates in the dogs subjected to IRE showed relatively wide gaps in glandular cavities, along with hemorrhaging, necrotic changes, and edema. By contrast, histologic examination of the prostatic urethra and surrounding prostatic capsules showed no significant damage (Fig. 3b). Microscopic comparisons of IRE-ablated and control regions showed that basal cells and acinar cells in the ablated regions of the gland had lost cytoplasmic volume compared with control cells, which maintained the normal shape of the gland (Fig. 3c).

**Figure 4:**
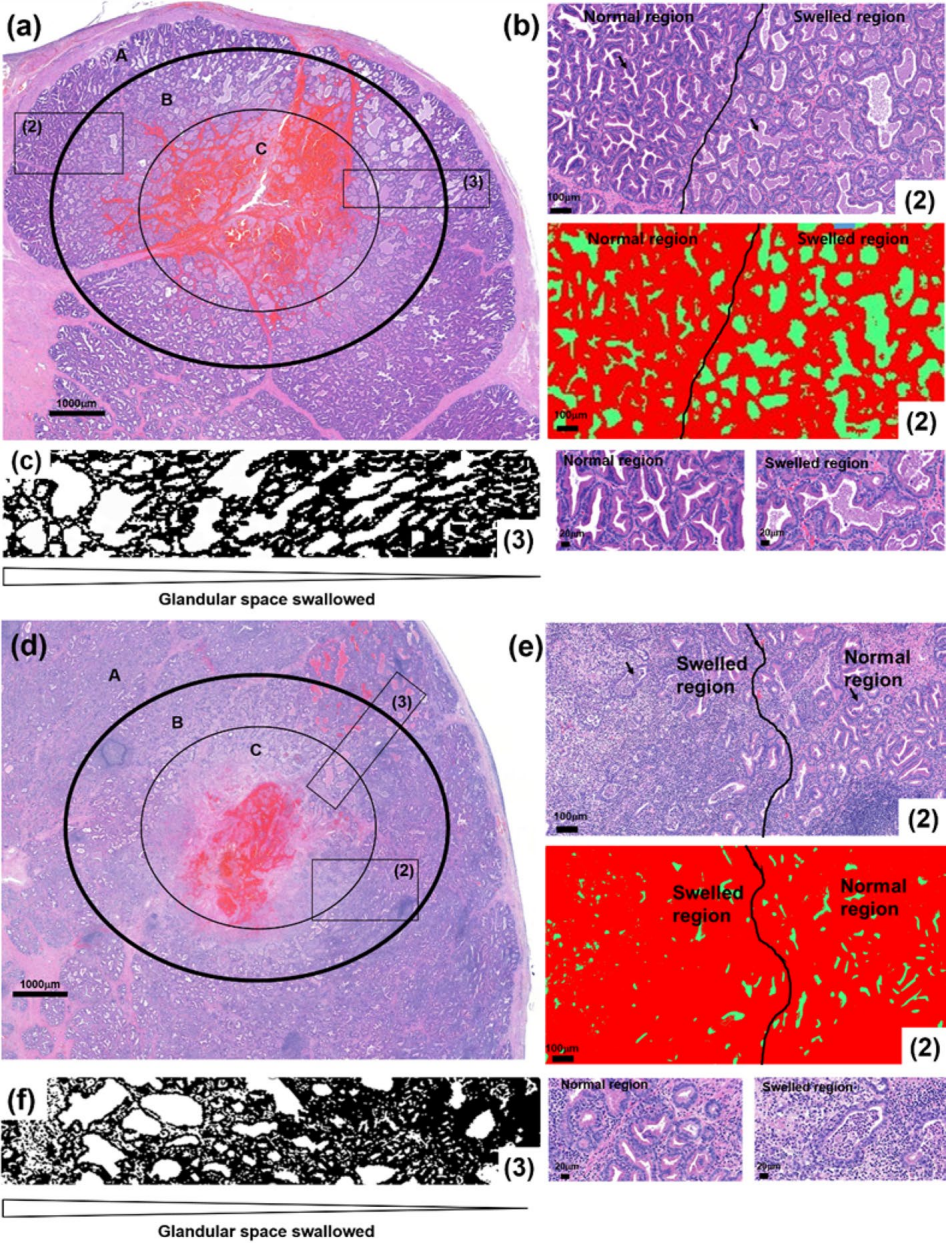
Effects of IRE on the shapes of prostate tissue. (**a**,**b**) Histologic examination, showing that normal (**A**), swollen (**B**), and completely ablated (**C**) areas of prostate tissue were not affected by (**a**) 500 and (**d**) 700 V. Demarcations between intact (1) and swollen (2) tissue are observed in (**a**) and (**e**). The acinar cells around the swollen glandular tissues were almost completely intact. The tendency to swell was dependent on the distance from the electrode (3) in (**a**) and (**d**).

The electrical fields applied to the prostate in this study are roughly proportional to the inverse-square of the distance^18^. Thus, these fields are reduced by the square of the distance from the anode or cathode to the tissue being ablated. Tissues affected by application of 500 and 700 V were divided into three regions: normal (A-region), swollen (B-region), and completely ablated (C-region) areas (Fig. 4). Glandular tissues in the B-regions experienced swelling (Fig. 4b,e), which decreased as the distance from the electrode increased (Fig. 4c,f). Cells distributed around the swollen glandular tissue were more intact than cells distributed around the glandular tissues in the A-region (Fig. 4b(3),e(3)).

**Figure 5:**
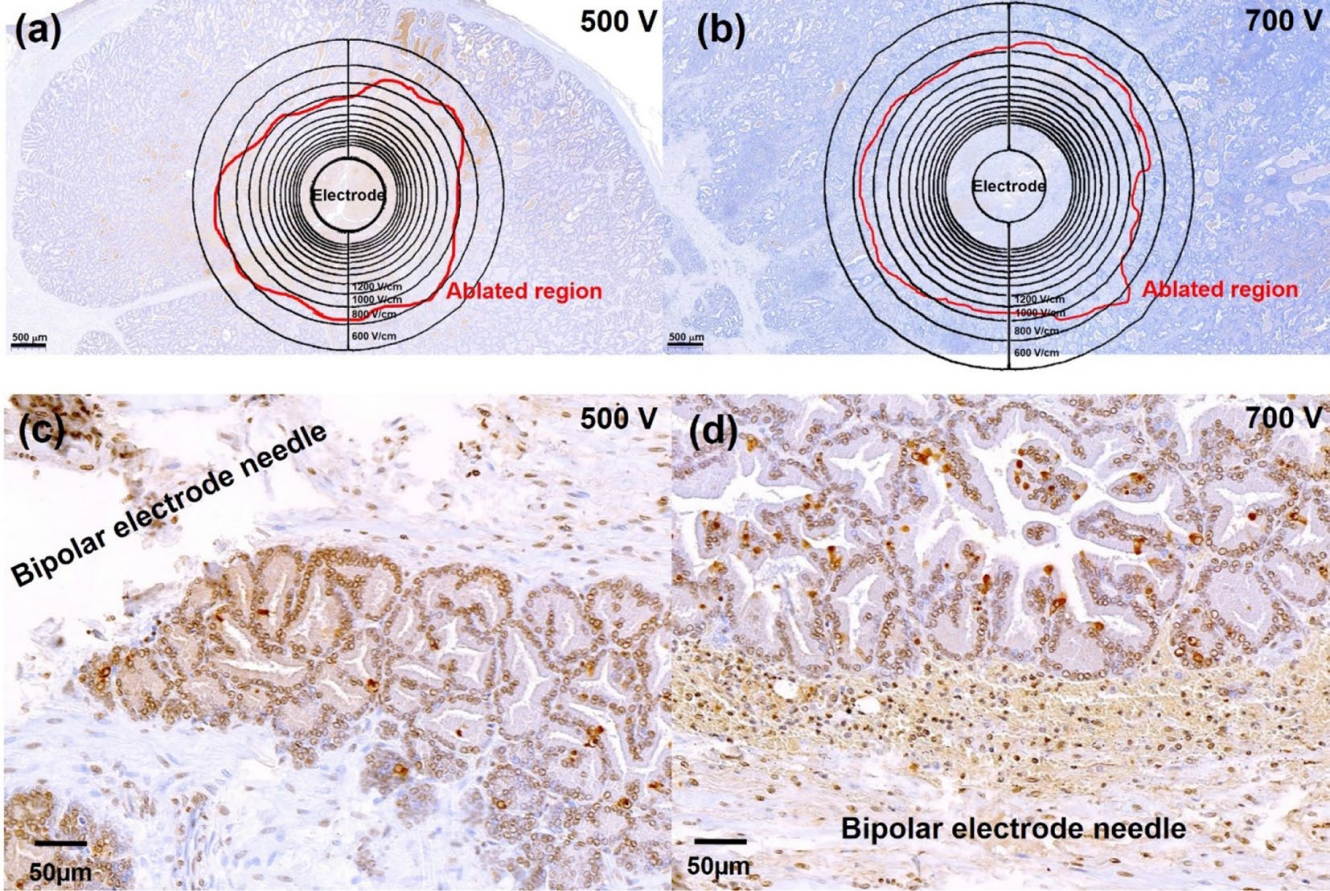
Microscopic images of TUNEL-stained slices following exposure to IRE at applied voltages of 500 V and 700 V. (**a**) Superposition of complete ablation, as determined by TUNEL assays, with isoelectric field strength curves in vertical sections of prostate tissues subjected to IRE at (**a**) 500 V and (**b**) 700 V. (**c**,**d**) TUNEL positive deposition following exposure to IRE at both (**c**) 500 V and (**d**) 700 V.

The mean (± standard deviation) areas of hemorrhage in the proximal (13.8 mm^2^ vs. 10.1 mm^2^), middle (8.8 mm2 vs. 7.1 mm^2^), and distal (5.4 mm2 vs. 5.8 mm^2^) sections were larger in tissues exposed to 500 V (9.3 ± 4.2 mm^2^) than 700 V (7.6 ± 2.2 mm^2^). TUNEL staining of tissues of the dogs exposed to IRE indicated that intense ablation was caused by irreversible damage to extra-normal tissues (Fig. 5). The areas ablated of 500 and 700 V were estimated to be 0.78 cm^2^ and 1.21 cm^2^, respectively (Fig. 5a,b). TUNEL positive deposition was strong in prostates of both dogs exposed to IRE (Fig. 5c,d).

## Discussion

IRE has several advantages in the treatment of prostate cancer, preserving the extracellular matrix, including the major vasculature and ductal systems and the neurons, thus minimizing the need for these structures to regenerate. Moreover, because the ablation site is far from the heart, IRE does not cause arrhythmias^19^. In conventional IRE, tissue is ablated by passing an electric current through multiple monopolar electrodes. In treatment of the prostate, at least one pair of electrodes is transperennially inserted into the prostate tissue undergoing ablation. These electrodes, however, cause skin perforations, resulting in bleeding. Moreover, parallel electrodes are required to make the electric field distribution even, thereby maximizing the ablation. Insertion of electrodes for IRE therefore required skilled operators.

Compared with monopolar electrodes, bipolar electrodes can reduce scarring and tumor invasiveness^20^. Moreover, skilled operators are not required to align electrodes parallel to each other, as only one bipolar electrode is inserted. Bipolar electrodes consist of a rod with the cathode and anode separated and axially. Increased separation between the anode and cathode can increase the region of ablation. In general, the mean prostate cancer tumor volume ablated during IRE is about 6 cc^21,22^. The length of the anode and cathode required to adequately cover the region to be removed is dependent on the volume of the tumor, despite the electric field strength thresholds being different in patient tissue. Multiple bipolar electrodes may be required to ablate larger prostate tumors. The present study tested the technical feasibility of the bipolar electrode by applying a single bipolar electrode to normal prostate tissue in beagle dogs. Our results indicated that IRE with a single bipolar electrode was technically feasible and safe when applied to the beagle prostate.

This study also found that the bipolar electrode could be easily inserted into the targeted site of the prostate under ultrasonographic guidance. Before applying IRE, it was necessary to select an electric field strength that does not cause burning, which can result from the passage of too high a current between electrodes. Electric field distributions of the applied 500 and 700 V were simulated and evaluated. While observing the current waveform on the oscilloscope, we tested the results of simulations on prostate tissue to determine whether 500 and 700 V cause electrical sparks between electrodes. When 700 V was applied through the bipolar electrode, the average current density was 21.04 A/cm^2^, resulting in no sparking, while 900 V overcurrent (Supplementary Fig. 1b). This current was below the average current density of 30.3–59.14 A/cm^2^ obtained by applying a monopolar electrode to localized prostate cancer^23^. This high current can result in electrochemical corrosion on the electrodes despite the applied pulsed width being very short. Oxidation occurs due to loss of electrons at the anode and reduction due to gain of electrons at the cathode. Anodic corrosion reveals the active or passive behavior of a metal, passive behavior indicating that the metal was more resistant to corrosion^24,25^. This was also indicated by the darker color of the electrode surface. In this study, active behavior was observed at the stainless steel electrode, not at the platinum electrode, depending on the coloration.

Each color in the simulated electric field distribution for the bipolar electrode represents an individual electric isofield contour. Ablation was therefore analyzed for applied 500 and 700 V. Although macroscopic analysis found that the ablated area of the prostate was larger when subjected to 500 V than to 700 V, histological analysis using TUNEL assays showed that the area ablated by applying 700 V was about 1.6 times larger than the area ablated by 500 V. This discrepancy may have been due to the greater degree of hemorrhage in the animal treated with 500 V, with the resulting necrosis-like tissues appearing black.

Hemorrhage is defined as chronic bleeding from an injured blood vessel^26^. In IRE, hemorrhage can be caused by both electric fields and their resulting currents. Unlike the field distribution of paired monopolar electrodes^10^, the field distribution of bipolar electrode has a dumbbell shape with a field strength that varies at different sites, with a maximum surrounding either the anode or cathode. To evaluate maximal ablation by IRE, sections surrounding the anode and cathode were evaluated histologically. Maximal ablation following application of 500 and 700 V was observed in the proximal and middle regions of the prostate, respectively. To determine the minimum electric field threshold required to induce IRE, their maximal ablations were matched to simulated electric field distributions. TUNEL assays showed that ablation induced apoptosis at electric field strengths over 800 V/cm following application of IRE at both 500 V and 700 V, suggesting that an electric field of 800 V/cm may be the minimum required for induction of IRE. This finding was greater than that for other tissues, such as 948 V/cm for cancerous prostate^19^ and 637 V/cm for healthy liver^27^. This discrepancy may have been due to differences in electrode geometry or in differences in the susceptibility of prostate tissues to IRE^28^.

Electric current can also affect hemorrhaging. The current between electrodes within prostate tissues flows randomly, depending on the impedance of the tissue. Changes in glandular tissues may be used to evaluate current flow. Normal glandular tissues were not observed in regions through which current passed, including swollen glandular zones within swollen regions and intact zones within demolished regions. This tendency was also observed following application of 700 V. Because the threshold of electric field distribution was 800 V/cm, glandular swelling may not be caused by the flow of the electric field but by electric current passing through a swollen region. Passing current can induce glandular swelling via a domain effect, which may behave in a manner consistent with spatial pattern dynamics. These dynamics behave thermodynamically as ensemble states, destroying in-folded structures. This pattern was more pronounced when prostate tissue was exposed to an electric field of 500 V than to an electric field of 700 V, and may have been due to higher electrical current flow, resulting in increased hemorrhaging. Furthermore, unfolded glandular swellings were found to be filled with tissue, whereas normal tissues were empty. Unruptured, unfolded tissues are surrounded by intact acinar cells, which were not stimulated by electrical current flow, in contrast to intact control cells. These cells may mediate androgen function that contributes to the ejaculate^29^.

The present study had several limitations, including the use of very few animals, thus preventing statistical determination of the effects of using the bipolar electrode for IRE in the beagle prostate model. Moreover, outcomes could not be compared in dogs that underwent prostate IRE using bipolar and monopolar electrodes. Besides, the dynamic conductivity term was not included in this electric field distribution simulation for convenience of calculation like other studies^30–32^. Nonetheless, this study suggested that an electric field strength of 800 V/cm was the threshold for IRE of the beagle prostate using the bipolar electrode. Moreover, this study provides understanding of the mechanism by which electrical current flow surrounding the electrodes causes swelling of glandular tissues.

The present study investigated the efficacy and technical feasibility of a newly developed bipolar electrode for IRE of the beagle prostate. This electrode could be easily inserted into beagle prostate tissue, with applying 500 V and 700 V, corresponding to electrical currents of 1.75 ± 0.25 A and 2.22 ± 0.35 A, respectively, resulting in the ablation of areas of 0.78 cm^2^ and 1.21 cm^2^, respectively. Moreover, the minimum electric field threshold required to induce IRE was estimated to be 800 V/cm. The IRE procedure using the bipolar electrode was technically feasible and safe in the beagle prostate. Non-thermal localized ablation using a single, novel bipolar electrode may be a promising therapeutic option for the treatment of prostate cancer, resulting in fewer procedure-related complications with an optimal ablation range.

## Supplementary Information

Supplementary Figures.
