# Supplementary material for: Novel platinum bipolar electrode for irreversible electroporation in prostate cancer: preclinical study in the beagle prostate
Source: Sci Rep. 2021 Aug 25;11:17194. doi: 10.1038/s41598-021-96734-5 (PMC8387373; doi:10.1038/s41598-021-96734-5)
Supplement: Supplementary file 1 — Supplementary Figures. [file 41598_2021_96734_MOESM1_ESM.docx]

**Supplementary Materials**

**Novel Platinum Bipolar Electrode for Irreversible Electroporation for Prostate Cancer: Preclinical Study in the Beagle Prostate**

Bumjin Lim, M.D.^1†^, Hongbae Kim, Ph.D.^2†^, Seung Jeong, M.S.^2,3^, Song Hee Kim, B.S.^4^, Jeon Min Kang, B.S.^4^, Yubeen Park, B.S.^4^, Dong-Sung Won, B.S.^4^, Ji Won Kim, B.S.^4^, Dae Sung Ryu, B.S.^4^, Yunlim Kim, Ph.D.^1^, Jung-Hoon Park, Ph.D.^4*^, Choung Soo Kim, M.D., Ph.D.^1*^

*^1^Departments of Urology, Asan Medical Center, University of Ulsan College of Medicine, 88 Olympic-ro 43-gil, Songpa-gu, Seoul, 05505, Republic of Korea*

*^2^Department of Biosystems & Biomaterials Science and Engineering, Seoul National University, Seoul, 08826, Republic of Korea*

*^3^Medical Engineering Research Center, The Standard Co. Ltd., Gyeonggi-do, 15880, Republic of Korea*

*^4^Biomedical Engineering Research Center, Asan Institute for Life Sciences, Asan Medical Center, 88 Olympic-ro 43-gil, Songpa-gu, Seoul, 05505, Republic of Korea*

^†^B.L. and H.K. contributed equally to this work and are the co-first authors.

^*^J.-H.P. and C.S.K. contributed equally to this work and are the co-corresponding authors.

**Correspondence:**

Jung-Hoon Park, Ph.D.^1^ and Choung Soo Kim, M.D., Ph.D.^2^

^1^Biomedical Engineering Research Center, Asan Institute for Life Sciences, Asan Medical Center, 88 Olympic-ro 43-gil, Songpa-gu, Seoul, 05505, Republic of Korea

Tel: 82-2-3010-4123 Fax: 82-2-476-0090

E-mail: jhparkz[@amc.seoul.kr](mailto:hyjung@amc.seoul.kr)

^2^Department of Urology, Asan Medical Center, University of Ulsan College of Medicine, 88 Olympic-ro 43-gil, Songpa-gu, Seoul 05505, Republic of Korea

Tel: 82-2-3010-3193 Fax: 82-2-476-0090

E-mail: cskim@amc.seoul.kr


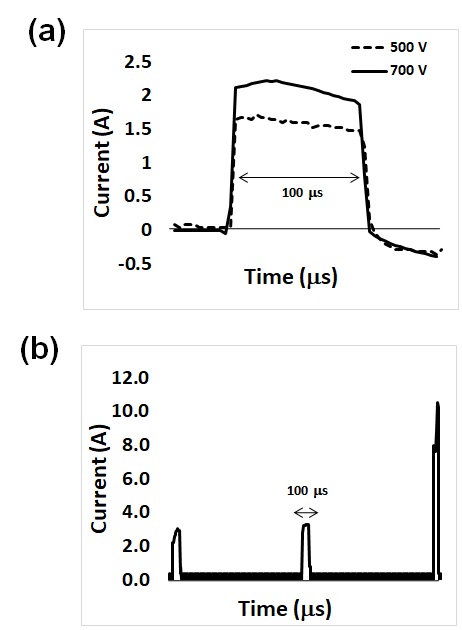


**Supplementary Figure 1.** Current waveforms corresponding to electrical currents of (a) 500 V and 700 V without sparks and (b) 900 V with sparks.


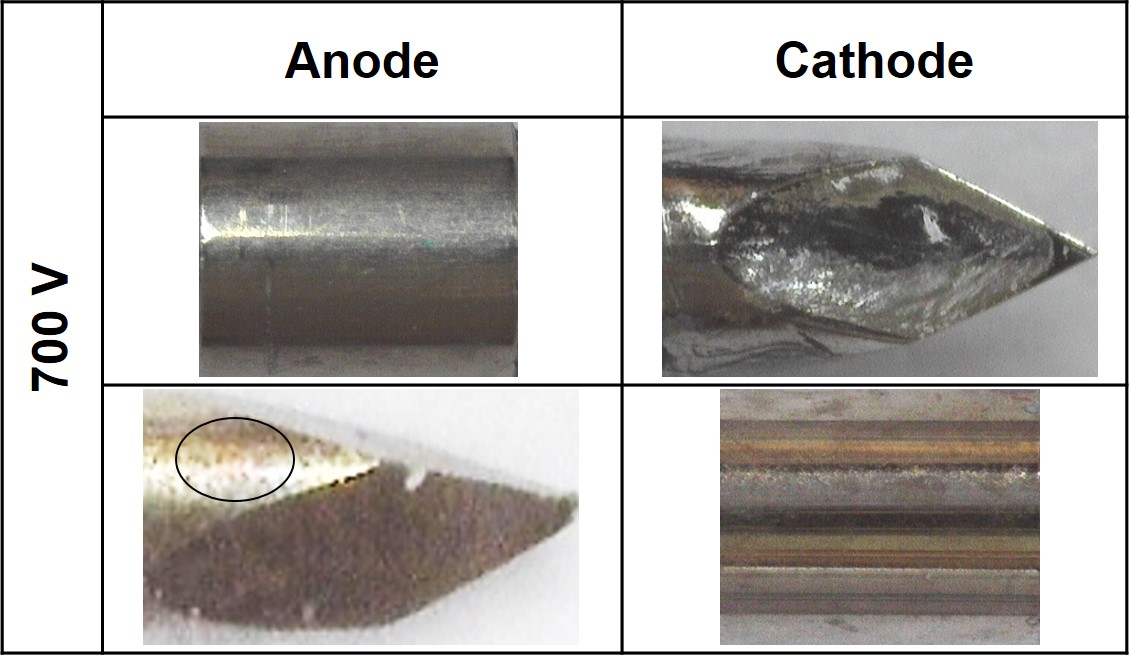


**Supplementary Figure 2.** Anodic corrosion of the platinum and stainless steel electrodes following application of 700 V. The platinum and stainless steel electrodes were alternated as anode and cathode to corroborate the corrosion. The darker circle on the anode indicates corrosion of the stainless steel electrode.


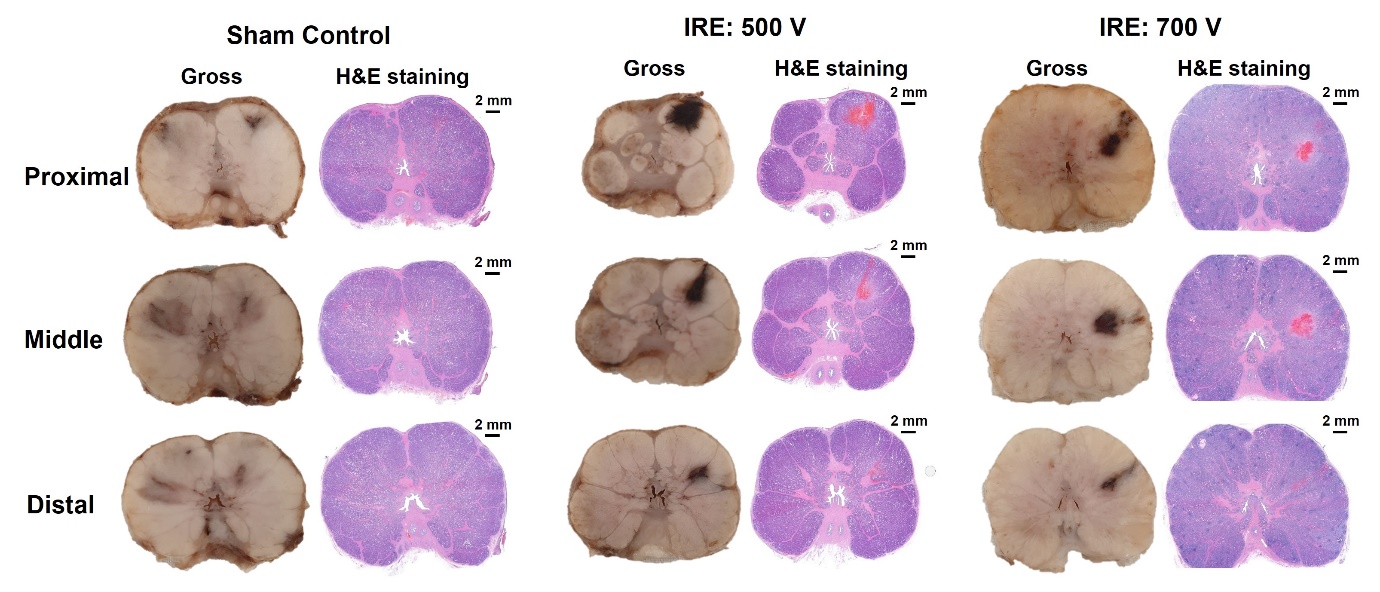


**Supplementary Figure 3.** Photographs of gross prostate specimens and histologic images of H&E-stained prostate tissue specimens of the prostate glands of the three beagle dogs.
